# Plastome Sequences Uncover the Korean Endemic Species *Polygonatum grandicaule* (Asparagaceae) as Part of the *P*. *odoratum* Complex

**DOI:** 10.3390/genes16040398

**Published:** 2025-03-29

**Authors:** Joonhyung Jung, Hyuk-Jin Kim, Joo-Hwan Kim

**Affiliations:** 1 Division of Forest Biodiversity, Korea National Arboretum, Pocheon 11186, Republic of Korea; jins77@korea.kr; 2 Department of Life Sciences, Gachon University, Seongnam 13120, Republic of Korea

**Keywords:** *Polygonatum grandicaule*, Korean endemic species, plastid genome (plastome), comparative phylogenomic analyses

## Abstract

**Background/Objectives:** *Polygonatum grandicaule* Y.S.Kim, B.U.Oh & C.G.Jang (Asparagaceae Juss.), a Korean endemic species, has been described based on its erect stem, tubular perianth shape, and pedicel length. However, its taxonomic status remains unclear due to limited molecular data. **Methods:** This study presents the complete plastid genomes (plastomes) of two *P. grandicaule* individuals and its close relative, *P*. *odoratum* (Mill.) Druce var. *thunbergii* (C.Morren & Decne.) H.Hara. **Results:** The plastomes, ranging from 154,578 to 154,579 base pairs (bp), are identical to those of *P*. *falcatum* A.Gray, *P*. *odoratum* var. *odoratum*, and another Korean endemic species, *P*. *infundiflorum* Y.S.Kim, B.U.Oh & C.G.Jang. All contain 78 plastid protein-coding genes (PCGs), 30 tRNA genes, and four rRNA genes, except for the pseudogene *inf*A. Phylogenetic analyses using 78 plastid PCGs and whole intergenic spacer (IGS) regions strongly support the three sections within *Polygonatum* Mill. and show that *P*. *odoratum* and its variety are nested within *P*. *falcatum*, *P*. *grandicaule*, and *P*. *infundiflorum*. **Conclusions:** Given the limited genomic variation and phylogenetic relationships, we propose treating *P*. *falcatum*, *P*. *grandicaule*, and *P*. *infundiflorum* as part of the *P*. *odoratum* complex, despite their morphological differences. This study offers valuable putative molecular markers for species identification and supports the application of plastome-based super-barcoding in the morphologically diverse genus *Polygonatum*.

## 1. Introduction

Species delimitation is fundamental in various fields of biology [1], especially when studying endemic plants that are restricted to specific regions. These plants are essential for understanding the evolutionary history of local flora and play a vital role in maintaining regional biodiversity [2,3]. Moreover, they provide valuable opportunities for discovering and conserving plant resources with significant economic potential [4]. Earlier studies on species delimitation primarily relied on morphological variation, often leading to ambiguous identification and classification. However, recent approaches combine both morphological and molecular data, including complete plastid genome (plastome) sequences, to more clearly define species boundaries [1,5,6].

In South Korea, 373 endemic taxa from 179 genera and 64 families have been recorded [7]. Among these, two species of *Polygonatum* Mill. (Asparagaceae: Nolinoideae: Polygonateae), *P*. *grandicaule* Y.S.Kim, B.U.Oh & C.G.Jang and *P*. *infundiflorum* Y.S.Kim, B.U.Oh & C.G.Jang, have been identified [8,9]. The genus *Polygonatum*, which contains approximately 80 species and is commonly known as Solomon’s seal, is the largest in the tribe Polygonateae and is widely distributed throughout the northern hemisphere [10,11]. Its rhizomes are used in traditional medicine, and their chemical components, biological activities, and pharmacological properties have been well-documented [12,13,14,15]. It is classified into three distinct sections based on phyllotaxis, basic chromosome number, and molecular evidence: (1) section *Polygonatum*, which includes species with an alternate phyllotactic pattern and a basic chromosome count of *x* = 9–11; (2) sect. *Sibirica*, which comprises species with a verticillate phyllotactic pattern and a basic chromosome count of *x* = 12; and (3) sect. *Verticillata*, which features species with diverse phyllotactic patterns and a basic chromosome count of *x* = 13–15 [16]. In South Korea, *P*. *grandicaule* is distinguished by its thick rhizome, cylindrical erect stem, nonbracteate type, tubular perianth shape, and glabrous, cylindroid (slightly S-shaped) filament [9]. It was classified in sect. *Polygonatum* based on alternate phyllotaxis [17]. However, research on *P*. *grandicaule* remains limited, with only partial genetic data available, and it is still unclear whether its diagnostic morphological traits are reliable and taxonomically informative.

Due to its crucial role in plant photosynthesis, researchers frequently use the plastome for phylogenetic reconstruction, estimating divergence times, and conducting biogeographical studies [18,19]. It is also commonly employed for species delimitation, providing valuable molecular markers based on genetic differences [20,21,22]. In general, it has a conserved structure composed of four regions: the large single-copy (LSC), the small single-copy (SSC), and two inverted repeats (IRs) [23]. Recent studies have employed complete plastome sequences to identify structural variations, determine phylogenetic positions and distribution patterns, and resolve ambiguous relationships arising from extensive morphological variation within this genus [24,25,26,27,28,29,30]. Another Korean endemic species, *P*. *infundiflorum*, has also been reported [31]; however, no plastome data for *P*. *grandicaule* are available. To clarify the status of the Korean endemic *Polygonatum* species, which have been identified solely on the basis of morphological characteristics, we collected two individuals of *P*. *grandicaule* and one individual of *P*. *odoratum* (Mill.) Druce var. *thunbergii* (C.Morren & Decne.) H.Hara from their natural habitats and sequenced their complete plastomes. These plastome sequences provide evidence to determine if the species is indeed endemic, while our comparative plastome structural study and phylogenetic analyses further substantiate its classification as a Korean endemic species. This study also provides valuable information on repeats and molecular diagnostic characteristics (MDCs), which can be used as future molecular identification markers.

## 2. Materials and Methods

### 2.1. Taxon Sampling, DNA Extraction, and Plastome Assembly

We collected two individuals of *P*. *grandicaule* from Yeongdong, Chungcheongbuk-do (36°17′42.6″ N, 27°48′07.5″ E) and Yeongwol, Gangwon-do (37°11′52.6″ N, 128°20′47.6″ E), as well as one *P*. *odoratum* var. *thunbergii* from Danyang, Chungcheongbuk-do (36°55′48.8″ N, 128°21′06.4″ E), in South Korea. All taxa were collected in sunny grasslands. After collection, voucher specimens were prepared for each sample and deposited in the Gachon University Herbarium (GCU), with unique accession numbers assigned to each (Table 1). Total genomic DNA (gDNA) was isolated following a modified 2 × CTAB extraction protocol [32]. The gDNA was then used for next-generation sequencing (NGS) on an Illumina Mi-seq platform (LAS, Seoul, Republic of Korea). Raw sequencing data were processed for de novo plastome assembly using the GetOrganelle toolkit [33]. A “map to reference” analysis was performed using Geneious Prime 2024.0.5 [34] to recheck and evaluate sequence coverage. Gene content and sequence order were annotated via GeSeq [35], and tRNAs were verified using the tRNAScan-SE web server (http://lowelab.ucsc.edu/tRNAscan-SE/ (accessed on 20 January 2025)) with default parameters [36]. Plastome visualizations were subsequently generated using the chloroplot web server [37].

### 2.2. Phylogenetic Analyses

We obtained 50 complete plastome sequences from National Center for Biotechnology Information (NCBI), including 33 from *Polygonatum* taxa spanning three sections, six from *Heteropolygonatum* M.N.Tamura & Ogisu taxa, five from *Disporopsis* Hance, and six from *Maianthemum* F.H.Wigg. (Table S1). The *Maianthemum* taxa were selected as the outgroup, given their basal position within the tribe Polygonateae as indicated by recent phylogenetic studies [29,38]. From these 53 taxa, 78 plastid protein-coding genes (PCGs) and intergenic spacer (IGS) regions were separately extracted, aligned with MUSCLE, and processed using Geneious Prime 2024.0.5 software [34].

To reconstruct the phylogenetic relationships within genus *Polygonatum*, we employed maximum parsimony (MP), maximum likelihood (ML), and Bayesian inference (BI) approaches. The MP analysis was carried out using PAUP* v4.0 [39], where all characters were treated as equally weighted and unordered, with gaps considered as missing data. We performed searches with 1000 replicates of random taxon additions and utilized tree-bisection-reconnection (TBR) branch swapping in PAUP*, retaining up to ten trees at each step. We conducted bootstrap analyses (parsimony bootstrap percentages, PBPs) with 1000 pseudoreplicates under the same parameters. ML analysis used the IQ-TREE web server (http://iqtree.cibiv.univie.ac.at/ (accessed on 2 March 2025)) incorporated the mean bootstrap percentage (MBP) and SH-like approximate likelihood ratio test (SH-aLRT) scores calculated over 10,000 ultrafast bootstrap replicates [40]. Before BI analysis, we determined the most suitable substitution model based on the Bayesian Information Criterion (BIC) in MEGA 11 (Table S2) [41]. BI was performed using MrBayes v3.2.7 [42] with two independent runs starting from random trees, each spanning at least 1,000,000 generations and sampled every 1000 generations. A 25% burn-in was applied, and the remaining trees were used to construct a 50% majority-rule consensus tree. Posterior probabilities (PPs) evaluated the BI tree’s robustness, ensuring effective sample size (ESS) values exceeded 200 for all parameters. Phylogenetic trees were finalized with FigTree v1.4.4 [43].

### 2.3. Nucleotide Diversity (Pi), Repeat Analyses, and Molecular Diagnostic Characteristics

Nucleotide diversity (*Pi*) of PCGs, tRNA genes, rRNA genes, and IGS regions was assessed for all *Polygonatum* taxa using DnaSP v6.0 [44] with a sliding window of 100 base pairs (bp) and a step size of 25 bp. Plastomes of all *Polygonatum* taxa were screened for simple sequence repeats (SSRs) using the MISA Perl script [45], with thresholds set at a minimum of 10 repeats for mononucleotides, 5 for dinucleotides, 4 for trinucleotides, and 3 for tetra-, penta-, and hexanucleotides. Additionally, REPuter [46] was used to detect forward, reverse, complementary, and palindromic repeats that were at least 30 bp in length and 90% similar, permitting a Hamming distance of 3. Furthermore, plastid PCGs were examined to identify MDCs unique to each genus in the tribe Polygonateae and to each of the three sections of the genus *Polygonatum*, using FastaChar v0.2.4 [47].

### 2.4. Relative Synonymous Codon Usage (RSCU) Analysis

We used DAMBE v7.3.11 [48] to analyze relative synonymous codon usage (RSCU) values in the 78 plastid PCGs of genus *Polygonatum* used in this study. Then, we used the pheatmap pacage in R (https://CRAN.R-project.org/package=pheatmap (accessed on 2 March 2025)) to construct the RSCU cluster diagram.

## 3. Results

### 3.1. Plastome Characteristics

**Figure 1:**
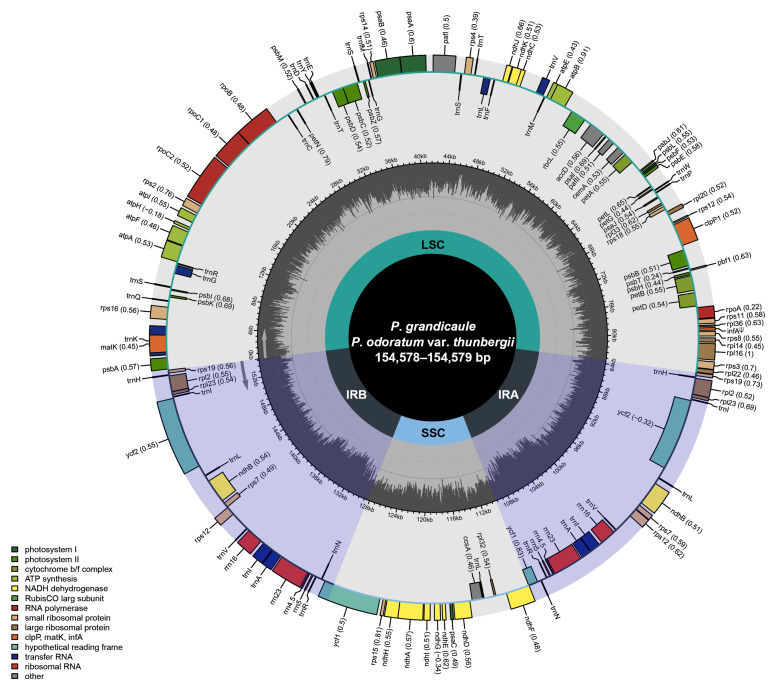
The complete plastome map of *P*. *grandicaule* and *P*. *odoratum* var. *thunbergii*, along with their gene contents. Colored boxes indicate conserved plastid genes. Genes inside the circle are transcribed clockwise, whereas those outside are transcribed counterclockwise. Additionally, the gray bar graphs in the inner circle show the GC content of the plastome.

A total of 24,720 to 109,056 reads were assembled, representing 0.89–4.87% of the total 8,807,104 to 11,204,918 reads generated (Table S3). The plastomes of two *P. grandicaule* individuals and one *P*. *odoratum* var. *thunbergii* individual exhibit a quadripartite structure, with a large single-copy (LSC) region (83,527–83,528 bp), a small single-copy (SSC) region (18,457 bp), and two inverted repeat (IR) regions (26,297 bp) (Figure 1 and Table 1). Across the three individuals, five point mutations were identified, with three located in non-coding regions and two within plastid PCGs (*rps*11 and *ycf*1). The plastome comprises 131 genes, including *inf*A identified as a pseudogene, along with 19 genes duplicated in the IR regions (Figure 1). In the analyzed sequence dataset, we identified 17 repetitive sequences. Fifteen of these sequences contain a single intron: *atp*F, *ndh*A, *ndh*B, *pet*B, *pet*D, *rpl*16, *rpl*2, *rpo*C1, *rps*16, *trn*A-UGC, *trn*G-UCC, *trn*I-GAU, *trn*K-UUU, *trn*L-UAA, and *trn*V-UAC. Only three sequences, namely *clp*P1, *paf*I, and *rps*12, contain two introns.

### 3.2. Phylogenetic Relationships

**Figure 2:**
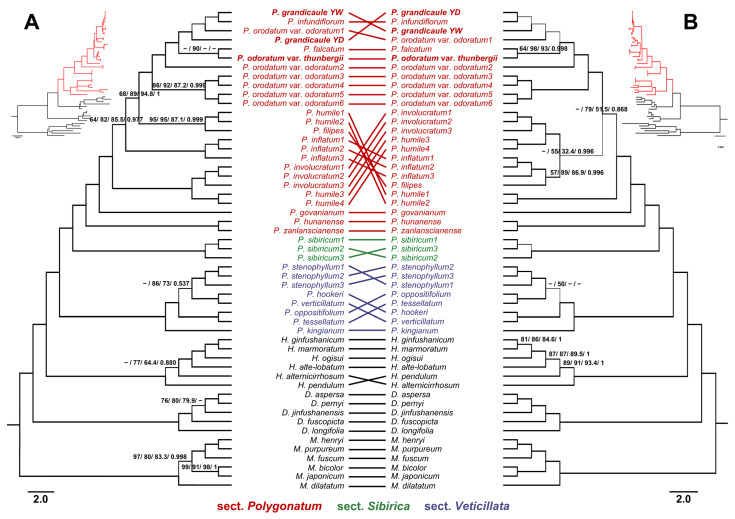
The maximum likelihood (ML) tree, constructed from (**A**) 78 plastid protein-coding genes (PCGs) and (**B**) whole intergenic spacer (IGS) regions, includes 53 taxa. Numbers indicate support values, represented as parsimony bootstrap percentages (PBPs)/mean bootstrap percentages (MBPs)/SH-aLRT support/posterior probability (PP). Only support values with MBP ≤ 90%, MBP ≤ 90%, SH-aLRT ≤ 90%, and PP ≤ 0.95 are displayed. Nodes with values below 50/50/50/0.5, or nodes exhibiting a different topology, are marked with “-”. The bold names indicate genomes obtained in this study. Abbreviations; YD: Yeongdong individual, YW: Yeongwol individual.

We performed MP, ML, and BI analyses, all of which produced phylogenetic trees with consistent topologies. These trees strongly supported the monophyly of the four genera within the tribe Polygonateae and three sections of genus *Polygonatum* (Figure 2). The alignment matrix of 78 plastid PCGs consisted of 68,433 characters, of which 66,539 (97.23%) were constant and 1236 (1.8%) were parsimony informative. The most parsimonious tree from this dataset was reconstructed with a tree length of 2361, a consistency index (CI) of 0.823, and a retention index (RI) of 0.941. Similarly, the IGS matrix comprised 44,488 characters, with 41,591 (93.49%) constant and 1870 (4.2%) parsimony-informative characters. The most parsimonious tree for the IGS dataset was reconstructed with a tree length of 4030, a CI of 0.753, and an RI of 0.910. Both results supported a sister relationship between *Heteropolygonatum* and *Polygonatum* (PBP = 100/MBP = 100/SH-aLRT = 100/PP = 1). Within *Polygonatum*, *P*. *humile* Fisch. ex Maxim. was determined to be non-monophyletic, while *P*. *falcatum* A.Gray, *P*. *grandicaule*, and *P*. *infundiflorum* clustered within the clade containing *P*. *odoratum* and its variety, forming what we refer to as the *P*. *odoratum* complex.

### 3.3. Comparative Plastome Sequences Analyses

**Figure 3:**
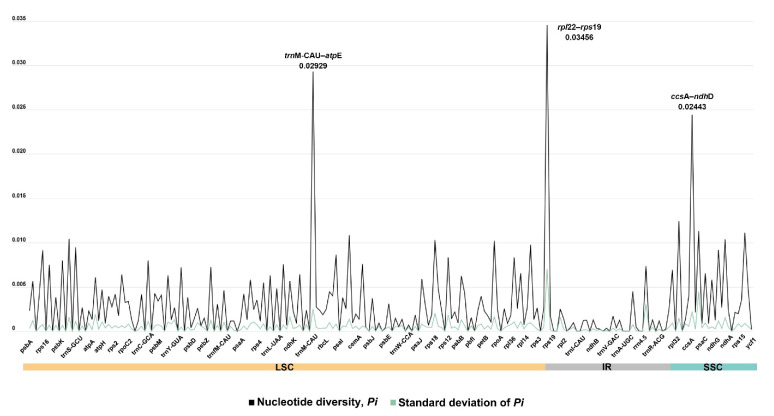
Nucleotide diversity (*Pi*) values of the plastomes of 36 *Polygonatum* taxa.

We examined nucleotide divergences in plastid PCGs, tRNA genes, rRNA genes, and non-coding regions to characterize variants among the 36 *Polygonatum* taxa (Figure 3 and Table S4). The nucleotide diversity (*Pi*) of plastid PCGs ranged from 0 (*pet*L, *pet*N, *psa*I, *psa*J, *psb*I, *psb*K, *psb*Z, *rpl*32, *rps*12, *rps*14, *rps*16, and *rps*7) to 0.00974 (*rpl*16), with an average value of 0.00175. In tRNA and rRNA regions, variations were detected in only five genes, with *Pi* values ranging from 0.0004 (*rrn*16) to 0.0242 (*trn*S-GCU). Among the non-coding regions, three IGS regions (*trn*M-CAU–*atp*E, *rpl*22–*rps*19, and *ccs*A–*ndh*D) exhibited notably high values (*Pi* > 0.02).

**Figure 4:**
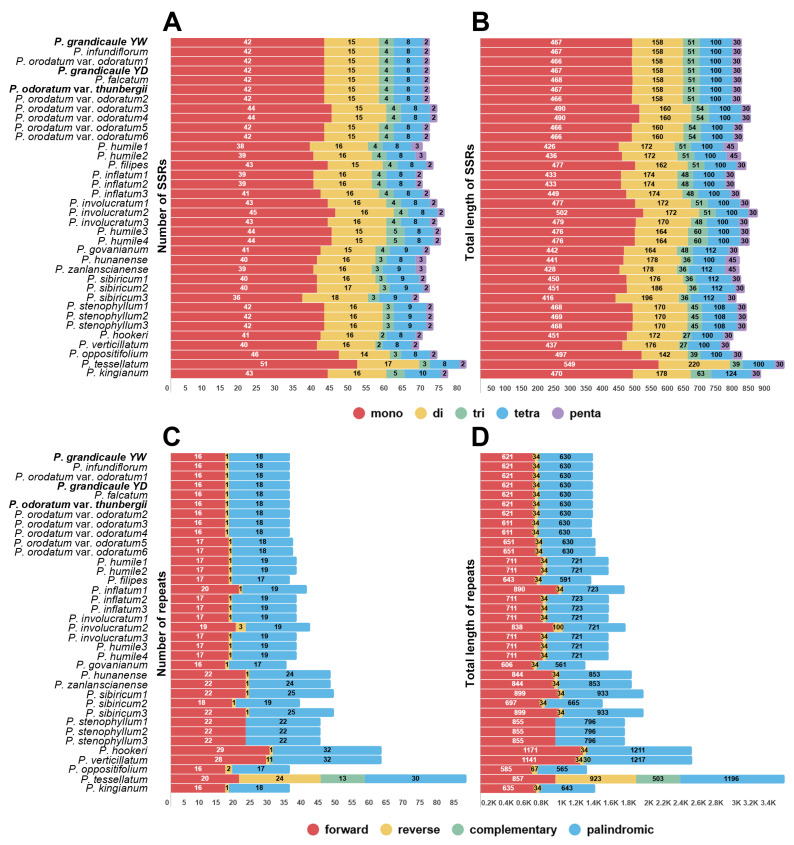
Statistics on different types of SSRs and repeats in *Polygonatum*. (**A**) The number of simple sequence repeats (SSRs) categorized by repeat unit length. “mono”, “di”, “tri”, “tetra”, and “penta” correspond to mononucleotide, dinucleotide, trinucleotide, tetranucleotide, and pentanucleotide repeats, respectively. (**B**) The total length of each SSR type. (**C**) The count of dispersed repeats, including forward, reverse, complementary, and palindromic repeats. (**D**) The cumulative length of the respective dispersed repeats. Abbreviations; YD: Yeongdong individual, YW: Yeongwol individual.

A total of 68–81 SSRs were detected in *Polygonatum* taxa, with a combined length of 770–938 (Figure 4). Within the *P*. *odoratum* complex, 71 SSRs were detected in *P*. *falcatum*, *P*. *grandicaule*, *P*. *infundiflorum*, four *P*. *orodatum* var. *odoratum*, and *P*. *odoratum* var. *thunbergii*, whereas 73 SSRs were found in two individuals of *P*. *odoratum* var. *odoratum*. Across these species, mononucleotide repeats predominated (42 and 44, respectively), accompanied by 15 dinucleotide repeats, 4 trinucleotide repeats, 8 tetranucleotide repeats, and 2 pentanucleotide repeats; no hexanucleotide repeats were observed. The total lengths ranged from 466 to 490 bp for mononucleotide repeats, 158 to 160 bp for dinucleotide repeats, 51 to 54 bp for trinucleotide repeats, 100 bp for tetranucleotide repeats, and 30 bp for pentanucleotide repeats. Most of the SSRs consisted of the A/T motif, while G/C motifs were comparatively less frequent (Table S5). Analysis of longer repeats revealed that, in *Polygonatum* taxa, forward and palindromic repeats occurred more frequently than reverse and complementary repeats, except in *P*. *oppositifolium*, which has 24 reverse and 13 complementary repeats and twice the total repeat length. Within the *P*. *odoratum* complex, 35 to 36 long repeats were identified, with only a single reverse repeat detected. Detailed information on the locations and frequencies of these longer repeats is provided in Table S6.

**Figure 5:**
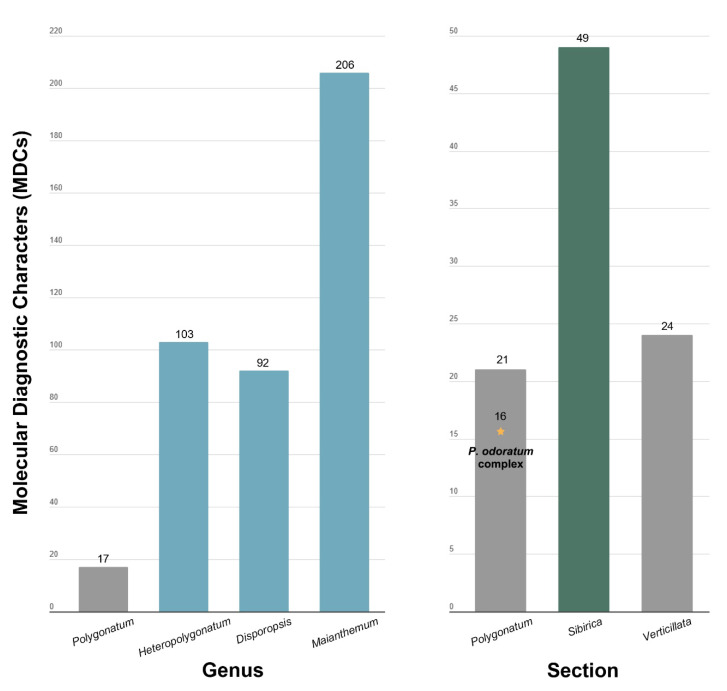
Quantitative analysis of molecular diagnostic characteristics (MDCs) in 78 plastid protein-coding genes (PCGs) across 53 Polygonateae species. The blue bars represent cases with more than 80 MDCs across genera, the green bars indicate cases with more than 30 MDCs within the genus *Polygonatum*, and the gray bars denote cases with fewer than 30 MDCs.

An alignment of 78 plastid PCGs from all members of the tribe Polygonateae identified 17 MDCs unique to the genus *Polygonatum*, 103 in *Heteropolygonatum* (including 42 deletions), 92 in *Disporopsis*, and 206 in *Maianthemum* (including 27 insertions and 9 deletions) (Figure 5 and Table S7). Within the genus *Polygonatum*, 21 MDCs were specific to the sect. *Polygonatum*, 49 to the sect. *Sibirica* (including 3 insertions), and 24 to the sect. *Verticillata* (including 9 insertions). It is important to note that this MDC analysis was conducted using an alignment that included all species within the tribe Polygonateae.

**Figure 6:**
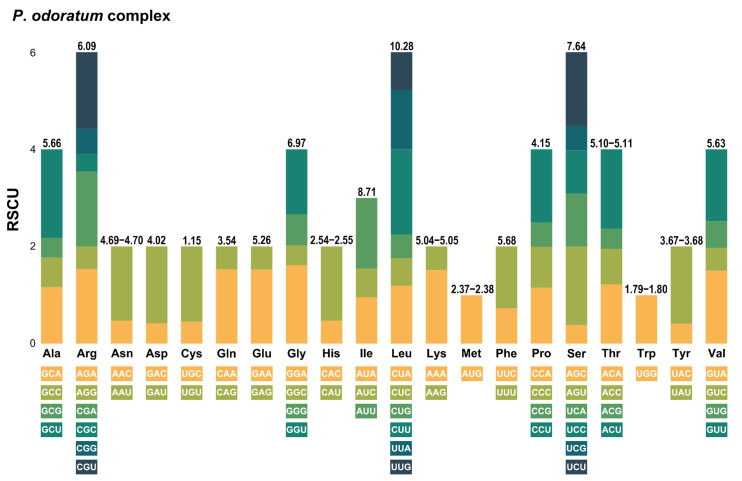
Relative synonymous codon usage (RSCU) analysis of 20 amino acids in 78 plastid protein-coding genes (PCGs) from the complete plastomes of the *P*. *odoratum* complex. The values at the top of each stacked bar indicate the usage frequency for each amino acid.

**Figure 7:**
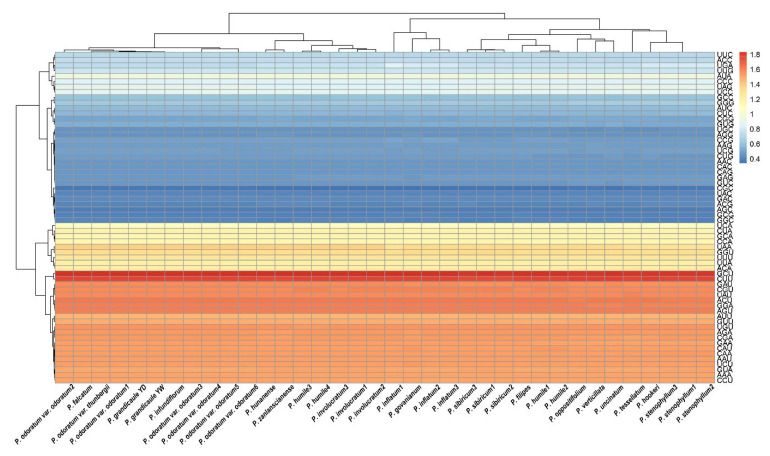
The heat map of codon usage bias in the plastomes of genus *Polygonatum*. The red color indicates higher relative synonymous codon usage (RSCU) values and the blue color indicates lower RSCU values. Abbreviations; YD: Yeongdong individual, YW: Yeongwol individual.

Using 78 plastid PCGs, we assessed the RSCU across all *Polygonatum* taxa (Figure 6 and Figure 7). *P*. *hookeri* exhibited the highest codon count with 22,662 codons, while *P*. *govanianum* had the lowest at 22,545 codons (Table S8). Leucine (L) was the most abundant amino acid, accounting for 10.26–10.30% of the codons, whereas cysteine (C) was the least frequent at 1.14–1.15%. Notably, about half of the codons had RSCU values above 1, indicating a preferential usage pattern that was consistent among species (Figure 7).

## 4. Discussion

### 4.1. The Characteristics of Plastomes in Polygonatum

In this study, we report the first complete plastomes for the Korean endemic species *P*. *grandicaule* and the closely related *P*. *odoratum* var. *thunbergii*. As noted in earlier research, the IR regions exhibit a higher GC content than both the LSC and SSC regions [24,25,26,27,28,29,30]. The *inf*A gene, which encodes the translation initiation factor 1 involved in assembling the initiation complex, was identified as a pseudogene across all *Polygonatum* taxa. To confirm its pseudogene status, the presence of an intact open reading frame (ORF) and a conserved domain was examined using the NCBI Conserved Domains Database (CDD), following Wang et al. [49]. The loss and pseudogenization of *inf*A have likewise been reported in other members of Asparagaceae [50,51,52]. In certain angiosperms, *inf*A has been shown to transfer from the plastome to the nuclear genome, where it is expressed and the resulting protein is imported back into the chloroplast [53,54]. This transfer preserves the essential function of *inf*A after the plastome copy becomes nonfunctional and demonstrates an evolutionary strategy in which organelle genes can migrate to the nucleus to ensure stable maintenance and regulation. During the verification process of plastome sequences, we confirmed that the nucleotide sequences of *P*. *falcatum*, *P*. *infundiflorum*, and one individual of *P*. *odoratum* var. *odoratum* already registered in NCBI are identical to those of *P*. *grandicaule* and *P*. *odoratum* var. *thunbergii* that we analyzed.

### 4.2. Taxonomic Ambiguity of Korean Endemic Polygonatum: A Phylogenetic Reassessment

Due to its complex morphological diversity, the phylogenetic relationships and intrageneric classification of *Polygonatum* have long been controversial. Earlier studies have reported non-monophyletic relationships among various *Polygonatum* species [16,24,25,26,27,28]. In our study, we reconstructed the phylogenetic relationships of *Polygonatum* alongside its closely related genera (*Heteropolygonatum*, *Disporum*, and *Maianthemum*) and found well-supported monophyletic relationships among these genera, which is consistent with previous findings [26,27,28,29,38]. However, several ambiguous relationships emerged among species within the sect. *Polygonatum*. Notably, *P*. *humile* exhibited a paraphyletic pattern, while *P*. *odoratum* and its variety, *P*. *odoratum* var. *thunbergii* were nested within a clade comprising *P*. *falcatum*, *P*. *grandicaule*, and *P*. *infundiflorum*, suggesting complex interrelationships among these taxa. Despite examining 78 plastid PCGs and the entire set of IGS regions, we detected minimal genetic variation among *P*. *falcatum*, *P*. *grandicaule*, *P*. *infundiflorum*, *P*. *odoratum* var. *odoratum*, and *P*. *odoratum* var. *thunbergii*, making it difficult to resolve their relationships with high confidence.

Research on *P*. *odoratum* has revealed extensive morphological and karyological diversity. For instance, an analysis of four Chinese cultivars revealed morphological differentiation and potential hybridization, as indicated by conflicting plastid and nuclear phylogenies [25]. Moreover, variation in ploidy levels and stem shape was observed, with some populations having quadrangular stems while others had cylindrical stems [55,56]. Given its broad distribution across Eurasia and significant trait variability among populations [10], a comprehensive worldwide study is needed to address both the morphological and molecular differences within this species.

The Korean endemic *Polygonatum* species were reported [8,9], and morphological differences between two endemic species and *P*. *thunbergii* C.Morren & Decne. were described [8]. Among the three species, a distinct feature of *P*. *grandicaule* is its erect stem and tubular perianth shape. Subsequently, Jang [17] classified the Korean *Polygonatum* species and highlighted key characteristics for their identification. *P*. *thunbergii* was identified by its thick rhizome, cylindrical stem, nonbracteate type, distinctive perianth shape, and glabrous, cylindroid (slightly S-shaped) filament. In contrast, Tamura [57] positioned *P*. *thunbergii* as *P*. *odoratum* var. *thunbergii*, noting that their stems are angled except at the base. Additionally, Jang [17] suggested that the key distinguishing characteristic separating *P*. *odoratum* (including its variety *P*. *odoratum* var. *pluriflorum* (Miq.) Ohwi) from *P*. *robustum* (Korsh.) Nakai, *P*. *grandicaule*, *P*. *infundiflorum*, and *P*. *odoratum* var. *thunbergii* is stem shape, which can be either quadrangular or cylindrical. Cytological studies of Korean *Polygonatum* species have shown that *P*. *grandicaule*, *P*. *infundiflorum*, and *P*. *odoratum* var. *thunbergii* share the same chromosome number (2*n* = 2*x* = 18), whereas *P*. *odoratum* differs (2*n* = 2*x* = 18 or 20) [58]. Since chromosome numbers can vary even within the same species [59], including *P*. *odoratum*, there is no issue in treating it as part of the *P*. *odoratum* complex. In previous palynological research, variables such as grain size, surface sculpturing, and pollen fertility were found to be highly variable, making them unsuitable as diagnostic characteristics for species recognition within the genus [60]. Recently, however, pollen shape has been found to be well-conserved, while pollen size and exine ornamentation are recognized as better identification characteristics in *Polygonatum* [61,62]. Further studies on palynological characteristics will aid in the revised classification of Korean *Polygonatum* species. Phylogenetic analyses of Korean *Polygonatum* using four plastidial loci also failed to resolve the positions of these taxa due to limited informative sites and identical sequences shared with *P*. *involucratum* (Franch. & Sav.) Maxim., *P*. *lasianthum* Maxim., *P*. *odoratum* var. *pluriflorum*, and *P*. *robustum* [63].

As mentioned earlier, the morphological characteristics used to classify each species, such as the presence or absence of an angular stem, stem curvature, filament length, and attachment position, should be re-evaluated when describing species delimitation in *Polygonatum*. Consequently, we propose that *P*. *falcatum*, *P*. *grandicaule*, *P*. *infundiflorum*, and *P*. *odoratum* var. *thunbergii* could be treated as part of a *P*. *odoratum* complex, despite their diverse morphological characteristics. Notably, individuals of Chinese *P*. *odoratum* were nested within *P*. *falcatum*, *P*. *grandicaule*, and *P*. *infundiflorum* in our phylogenetic analysis, raising further questions about the taxonomic status of two Korean endemic *Polygonatum* species. Hybrid capture with high-throughput sequencing (Hyb-Seq) analyses of 40 Korean *Polygonatum* taxa also revealed morphological ambiguity and classified both *P*. *grandicaule* and *P*. *odoratum* var. *thunbergii* as *P*. *odoratum* var. *odoratum*, which partially supports this treatment [64]. To establish a more comprehensive basis for the taxonomic revision of Korean *Polygonatum*, future research should incorporate a broader sampling of species and individuals, including those from different regions such as China.

Furthermore, phylogenomic studies based on nuclear genes have shown considerable discordance with plastid genome data in *Polygonatum* [38]. To build a more robust phylogenetic framework, expanded sampling across diverse habitats and the use of integrated genomic approaches, such as transcriptome and whole-genome sequencing, are essential.

### 4.3. Comparative Analyses and Putative Markers for Polygonatum

We identified *trn*M-CAU–*atp*E, *rpl*22–*rps*19, and *ccs*A–*ndh*D as notably divergent regions, each with a *Pi* value exceeding 0.02. Notably, the *ccs*A–*ndh*D locus aligns with previous findings [28]. Additionally, further research is needed to evaluate whether regions with a high *Pi* value may serve as effective markers for phylogenetic analysis and species identification.

SSRs are widely regarded for their high polymorphism and utility in genetic diversity assessments, molecular identification, and population genetics. In the present study, mononucleotide repeats composed mainly of A/T bases predominated and were primarily located in non-coding regions, mirroring patterns reported in other members of Asparagaceae [65,66]. Additionally, we detected extended repeats in *Polygonatum*, which showed minor variations among different repeat types. We also analyzed the MDCs in four genera of the tribe Polygonateae as well as in the genus *Polygonatum*. In the *P*. orodatum complex, a total of sixteen MDCs were identified across the tribe, including one each in *acc*D, *ndh*I, *psa*A, *psb*T, *rpo*A, *rpo*B, *rpo*C2, and *rps*15, and two each in *atp*B, *psa*B, *rpl*20, and *ycf*1. We observed that substitutions in *atp*B, *rpl*20, *rps*15, and *ycf*1 led to alterations in translation, resulting in changes in protein structure depending on whether the substitutions were conservative or non-conservative. Notably, all these genetic variations will enhance species identification in the morphologically diverse genus *Polygonatum*.

An analysis of codon usage in plastid PCGs underscores the influence of mutation trends and selective pressures at the species level. Overrepresented codons predominantly ended in A or U, while those with a lower frequency typically terminated in G or C. The codon AUU (Ile) was the most prevalent, consistent with patterns observed in other Asparagaceae species [67,68]. Overall, these results highlight the value of codon usage studies in elucidating evolutionary processes.

## 5. Conclusions

In this study, we examined the complete plastome sequences of the Korean endemic *P*. *grandicaule* and its close relatives. Our results support the monophyly of the genus *Polygonatum*, which can be divided into three sections based on the current sampling. Phylogenetic analyses also highlight the taxonomic complexity among the Korean endemic *Polygonatum* species. We propose that *P*. *falcatum*, *P*. *grandicaule*, and *P*. *infundiflorum* could be considered part of a *P*. *odoratum* complex, as *P*. *odoratum* individuals are nested within three species. Due to the limited genetic divergence among members of this complex, analyses of SSRs, dispersed repeats, and RSCU further support their close relationship. Additionally, we identified MDCs of members of the tribe Polygonateae, including those within the genus *Polygonatum*. These findings offer valuable resources for developing robust molecular markers and provide important insights for future super-barcoding research in this morphologically diverse genus. Although the current infrageneric classification of *Polygonatum* is generally accepted, intrageneric classification remains ambiguous, as it relies heavily on morphological traits in several species. To resolve these relationships more accurately, future studies should incorporate broader sampling across species and populations, using Hyb-Seq, transcriptome sequencing, and whole-genome sequencing.

## Figures and Tables

**Table 1:** Features of the LSC, SSC, and IR of plastomes obtained in this study.

**Taxa**	**Length and G+C Content**				**GenBank** **Accession No.**	**Voucher** **(Accession)**
	**LSC bp** **(G+C%)**	**SSC bp** **(G+C%)**	**IR bp** **(G+C%)**	**Total bp** **(G+C%)**		
*P. grandicaule* Y.S.Kim, B.U.Oh & C.G.Jang (Yeongdong, Chungcheongbuk-do)	83,527 (35.8)	18,457 (31.6)	26,297 (43.0)	154,578 (37.7)	PV199344	JH220610014
*P. grandicaule* Y.S.Kim, B.U.Oh & C.G.Jang (Yeongwol, Gangwon-do)	83,528 (35.8)	18,457 (31.6)	26,297 (43.0)	154,579 (37.7)	PV199345	JH220617027
*Polygonatum odoratum* var. *thunbergii* (C.Morren & Decne.) H.Hara (Danyang, Chungcheongbuk-do)	83,527 (35.8)	18,457 (31.6)	26,297 (43.0)	154,578 (37.7)	PV199346	JH220617013

## Data Availability

The three plastome sequences we obtained from this study were archived in NCBI. The accession numbers are presented in Table 1 (PV199344–PV199346).

## Supplementary Materials

The following supporting information can be downloaded at: https://www.mdpi.com/article/10.3390/genes16040398/s1, Table S1. List of species used for phylogenetic analyses. Table S2. Maximum Likelihood fits of 24 different nucleotide substitution models. Table S3. List of sampling taxa and plastome assembly information. Table S4. Nucleotide diversity (*Pi*) and standard deviation of *Pi* for 33 Polygonatum taxa. Table S5. The feature of SSRs in the complete plastomes of Polygonatum. Table S6. The feature of long repeats in the complete plastomes of Polygonatum. Table S7. The molecular diagnostic characteristics (MDCs) in the 78 plastid protein-coding genes. Table S8. Relative synonymous codon usage values of Polygonatum.
